# Disseminated hydatidosis an unusual presentation: a case report

**DOI:** 10.1099/acmi.0.000803.v3

**Published:** 2024-09-23

**Authors:** Fatima Ziad, Imane Zouaoui, Mostaine El Mamoune, Sarra Aoufi

**Affiliations:** 1 Central Laboratory of Parasitology-Mycology, Ibn Sina University Hospital, Rabat, Morocco; 2 Faculty of Medicine and Pharmacy, Mohamed V University, Rabat, Morocco

**Keywords:** disseminated hydatidosis, *Echinococcus granulosis*, Morocco

## Abstract

Hydatidosis, also known as cystic echinococcosis, is a widespread zoonosis, caused by a tapeworm of the genus *Echinococcus*. It presents a significant public health concern, particularly in endemic areas. The occurrence of disseminated hydatid disease is uncommon, even in regions where it is endemic, with an incidence ranging from 1–8%. The definitive diagnosis relies on a parasitological method.

In this work, we present an unusual case of disseminated hydatid disease that was diagnosed in the central parasitology–mycology laboratory of ‘The Ibn Sina University Hospital’.

This is a 21-year-old patient residing in a rural area, who presented with heaviness-type pain in the right hypochondrium, accompanied with nausea and vomiting. During the examination, the patient mentioned the contact with dogs. Abdominal radiography (ultrasound and CT) revealed findings suggestive of multiple hydatid cysts located in the liver and peritoneum. This suspicion was confirmed by positive hydatid serology.

After 9 months of treatment with albendazole, the patient underwent surgery for excision of the cysts shown on the x-ray, as well as other cysts incidentally discovered intraoperatively at the pelvic and rectal levels. All of the extracted specimens were sent to the parasitology laboratory. The direct examination, along with the viability test, revealed the presence of hooks and scolex of non-viable *Echinococcus granulosus*.

Disseminated hydatidosis is a rare but serious presentation, and the positive diagnosis relies on several epidemiological, clinical, radiological and parasitological arguments. Medical and surgical treatments play a crucial role in determining the patient’s prognosis.

## Data Summary

No data has been reused or generated in this work.

## Introduction

Hydatidosis or cystic echinococcosis poses a significant public health problem, particularly in endemic areas. It is a cosmopolitan zoonosis caused by a tapeworm of the genus *Echinococcus*, *Echinococcosis* occurs in four forms. The two most important forms, which are of medical and public health relevance in humans, are cystic echinococcosis (CE) also known as hydatid disease or hydatidosis, caused by infection with a species complex centred on *Echinococcus granulosus*; and alveolar echinococcosis (AE) caused by infection with *E. multilocularis* [1]. Disseminated hydatid disease is a rare clinical presentation, even in endemic regions, with an incidence ranging from 1–8% [2,3]. However, spontaneous rupture of the cysts accounts for 12% of the cases worldwide. Primary disseminated hydatid disease accounting for 2% of intra-abdominal hydatidosis [4,5]. The confirmatory diagnosis is parasitological. In this study, we report an unusual case of disseminated hydatidosis in a 21-year-old individual.

## Case presentation

This is a 21-year-old Moroccan patient residing in a rural area in the vicinity of Laarach (North of Morocco), with regular contact with his own dog for a year. The history of the disease dates back to 2010 marked by the onset of mild pain in the right hypochondrium.

In 2022, the patient was admitted in our hospital in the Vascular Surgery Department for sought medical attention due to an increase in the pain in the right hypochondrium accompanied by nausea and vomiting. Abdominal radiography [ultrasound (US) and computed tomography (CT)] revealed multiple hydatid cysts in the liver (four cysts in segments VII and VI, three of which were unilocular and one multilocular, measuring 105×90 mm, 83×31 mm, 43×28 mm and 30×19 mm) and peritoneum (the cysts were unilocular, five in the left iliac fossa, measuring approximately 30×24 mm, and one in the right iliac flank, measuring 25×38 mm). The cysts were classified as type IV according to the Gharbi classification [6], and type CE1, CE2 and CE3a according to the World Health Organization (WHO) classification [7].

Hydatid serology performed in a private laboratory in Laarach, using the indirect haemagglutination technique (IHA), was positive [the titre >1280 (the positivity threshold is ≥320)].

Medical treatment with albendazole (800 mg/day) was initiated and the patient was regularly monitored in consultation every 3 months in our hospital.

**Fig. 1.**
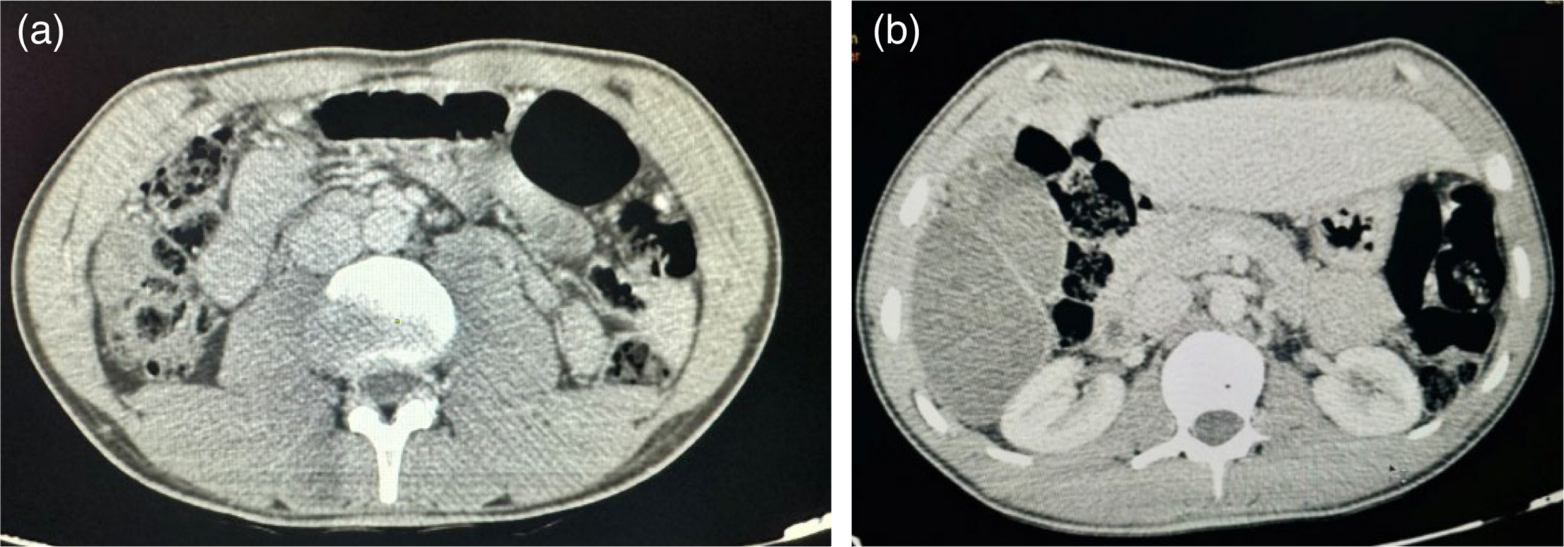
Abdominal CT scan (cross-section) showing a large hydatid cyst in the right lobe of the liver with peritoneal dissemination in the inter-hepato-phrenic space and along the Arantius groove.

After 9 months of daily medical treatment, a follow-up CT scan revealed a large hepatic hydatid cyst in the right lobe, spanning segments V, VI, VII and VIII, measuring 123×108×123 mm. Peritoneal dissemination was observed in the inter-hepato-phrenic space and along the Arantius groove (Fig. 1).

Clinically, the patient was stable with constant pain in the right hypochondrium, respiratory function was without abnormalities, and the chest x-ray performed at a private radiology centre was normal.

Laboratory parameters, liver function tests [aspartate transaminase (AST) and alanine transaminase (ALT)] and complete blood count showed no abnormalities (the eosinophil level was normal). Hydatid serology performed in our laboratory indicated a titre of 30.30 NTU [Novalisa Echinoccus IgG, ELISA (the positivity threshold is 11 NTU)].

Surgical treatment was proposed and a median laparotomy straddling the umbilicus was performed. Intra-operatively, multiple cysts of varying sizes were discovered, involving almost the entire right hepatic lobe, along with the gall bladder intact, the greater omentum, the mesentery, the small intestine, the pelvis, subhepatic and pre- and latero-rectal areas.

**Fig. 2.**
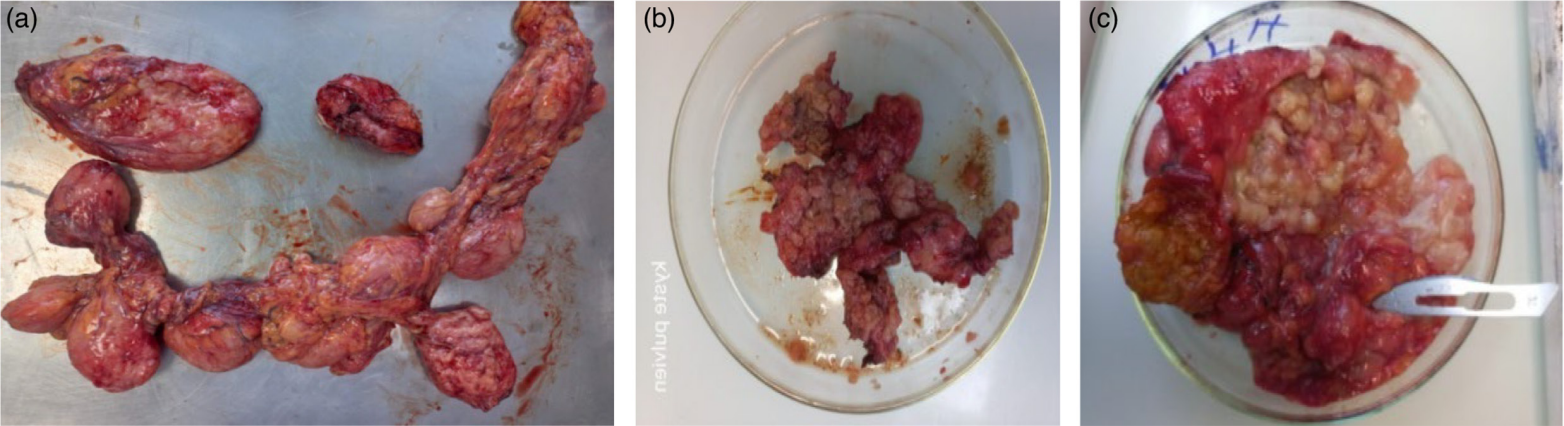
Macroscopic examination of the samples revealed several pieces of different sizes from various locations: (a) peritoneal, (**b**) pelvic and (c) hepatic.

All of the extracted surgical specimens were sent to the parasitology laboratory for examination. The macroscopic examination of the samples revealed several pieces of different sizes from various locations (Fig. 2).

**Fig. 3.**
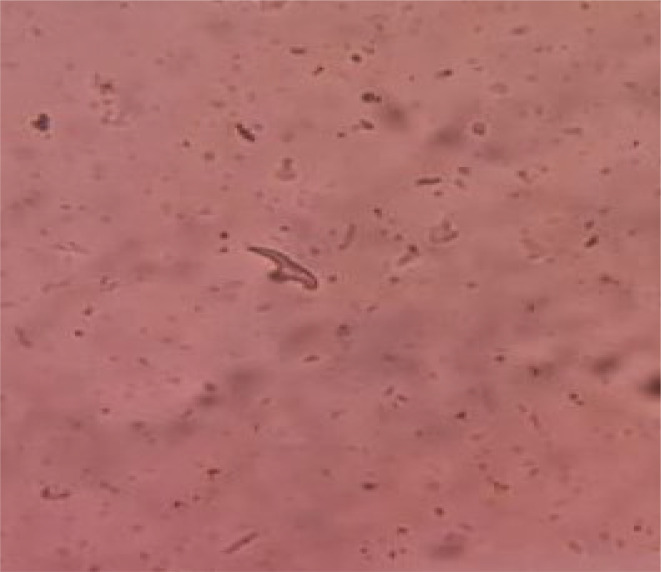
Microscopic examination showed hooks of *E. granulosus*.

Upon microscopic examination of the contents obtained by scraping the internal face of each cuticle, hooks of *E. granulosus* were identified at the pelvic part. Additionally, hooks, invaginated and devaginated scolex of this parasite were observed in the peritoneal and hepatic parts. The 0.2% eosin viability test indicated non-viable scolex of *E. granulosus* (Figs3  4).

**Fig. 4.**
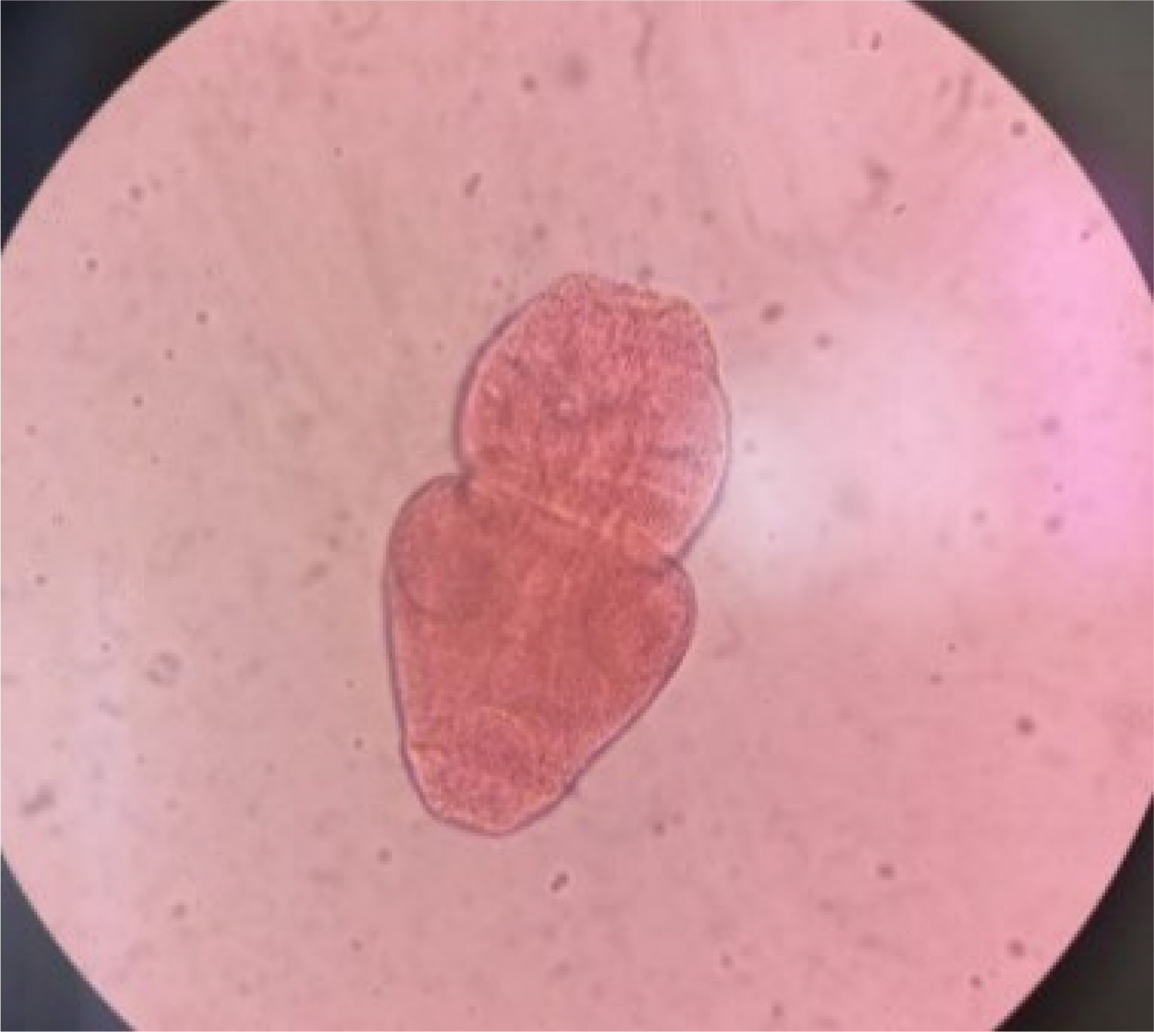
The 0.2% eosin viability test indicated non-viable scolex of *E. granulosus*.

All the epidemiological (endemic region, exposure with dog), clinical, radiological and parasitological data confirmed the diagnosis of disseminated hydatid disease.

After the operation treatment, medical treatment with albendazole (800 mg/day) was continued, and the patient was regularly monitored (biologically and clinically) every 3 months in our hospital.

## Discussion

Hydatidosis is endemic in various regions such as East and North Africa, Central Asia and China, with a prevalence rate of 5–10% [8]. Globally, the annual infection rate is approximately 1.2 million, resulting in a mortality rate of 2.2% per year [9]. In the literature, hydatidosis is commonly reported in young adults [10], and is particularly prevalent in regions where sheep and cattle are raised [11]. Morocco, specifically, is considered endemic for hydatidosis. The incidence is underestimated because only surgical cases are recorded [12]. In humans, the rate of 5.95 surgical cases per 100 000 inhabitants was recorded in 2018–2022, the frequency of CE was higher in women than in men, with 58.29 and 41.71%, respectively, and rural areas were significantly more affected than urban areas for both men and women. The distribution of cases by age groups: indeed 23.03% of patients were aged less than 18 years, 45% for 18–44 years aged group and 31.98% for over 45 years aged group. Overall, 6.90 % of surgical cases were reported in the northern region of Morocco [13]. The hepatic localization is the most frequent in several studies [13,14]. To our knowledge AE has not been reported in Morocco.

Contact with dogs stands out as the primary risk factor for human infection. Humans, being the accidental intermediate hosts, acquire the infection by ingestion of eggs present in contaminated food, water or soil, or through direct contact with dogs, which are the definitive hosts [3,9, 15]. CE prevalence in dogs ranges between 23.5 and 38.8% (owned dogs) and between 51.3 and 68.5% (stray dogs). In livestock, CE prevalence at slaughterhouses is 12.4% in cattle, 8.7% in camels, 8.4% in sheep and 4.7% in goat [12]. Our patient aligns with these findings, being a young individual from a rural region with constant exposure with dogs.

The preventive measures are focused on interrupting the parasite life cycle, controlling the dog population, and protecting livestock, principally by improving hygiene standards in slaughterhouses, deworming dogs, and controlling stray dogs. The Ministry of Agriculture and the Ministry of the Interior organize and supervise the activities related to this axis [12].

In humans, hydatid cysts are predominantly located in the liver in 50–75% of cases, with 25% found in the lungs [15,16]. Disseminated abdominal hydatid disease is an exceptional presentation, accounting for 5–16% of all locations combined according to European series. Primary dissemination is extremely rare representing 2% of intra-abdominal hydatidosis [3].

Intraperitoneal rupture of a hydatid cyst is an uncommon clinical presentation, even in endemic areas, with an incidence ranging from 1–8% [2], and causes serious problems and severe, life-threatening complications, including anaphylaxis [17]. This typically occurs following spontaneous or accidental rupture of hepatic or splenic cysts during surgery. The release of cystic fluid into the peritoneal cavity results in the development of multiple disseminated cysts, which causes abdominal distension, ascites and intestinal obstruction [2,18]. In this present observation, our patient has never undergone any previous surgeries and he presented with multiple hydatid cysts located in hepatic, peritoneal and pelvic.

Common clinical signs during this complication include abdominal pain, nausea and vomiting [2], and our case aligns with these reported symptoms.

The diagnosis of hydatidosis essentially relies on radiological examination and parasitological examination. Radiography serves as the initial examination, demonstrating a great sensitivity ranging from 95–100%, and aiding in determining the organ of origin and the characteristics of the cyst [2,10, 19]. In our country, hydatidosis is often discovered incidentally. In a Moroccan study during the screening campaigns, of 5221 subjects who received abdominal US, 132 people had at least one abdominal CE or suspect abdominal lesion. Of which, 102 subjects (1.9%) had at least one abdominal CE lesion, either a CE cyst (92.2%) or a residual lesion from previous abdominal CE surgery (7.8%) [20]. In the patient’s case, the radiology (US and CT) results were suggestive of disseminated hepatic and peritoneal hydatidosis.

Parasitological examination involves serology (ELISA, IHA and Western blot) and direct microscopic examination. Serology is crucial for confirming the hydatid nature of a suspicious radiological image, with significant predictive value [21]. Its sensitivity depends on factors such as the location, number, integrity, viability of the hydatid cyst and the assay technique. For instance, it reaches approximately 96% for hepatic hydatid infections, 74% for pulmonary hydatid infections and less for other locations [22]. In our case, hydatid serology was a strong positive result.

Microscopic parasitological examination serves as the confirmatory test, allowing for the study of the hydatid cyst contents, and the assessement of the scolexes viability [19]. This was confirmed in the case of our patient, where the direct microscopic parasitological examination of all surgical specimens revealed the presence of hooks and scolex of non-viable *E. granulosus*.

According to the WHO, the management of hydatid diseases involves several therapeutic approaches, including medical treatment with antihelmintics (albendazole, praziquantel or ivermectin), percutaneous aspiration, excision or watchful waiting [8].

The recommended dose of albendazole for adults is 15 mg/kg/day without exceeding 800 mg/day, administered in two doses. The duration of preoperative treatment varies from 1 to 6 months [23,24]. The results of albendazole treatment showed a cure rate of 50–70% at the 12-months follow-up [25]. This chemoprophylaxis aims to reduce intracystic pressure, facilitating its surgical excision and significantly limiting the viability of the scolex, thereby minimizing the risk of postoperative recurrence of hydatidosis [21]. In our case, it was the clinicians who decided on the dose and duration of albendazole.

The combination of surgery and albendazole therapy is a beneficial treatment approach. A preoperative diagnosis can guide the selection of the appropriate treatment, involving preoperative albendazole therapy to reduce intracystic pressure, preventing discharge into the abdominal cavity, and postoperative albendazole therapy to further ensure efficacy [26]. Our case adhered successfully the therapeutic protocol, as confirmed the examination of the surgical specimens in the laboratory by the identification of non-viable *E. granulosus* scolexes.

## Conclusion

Disseminated hydatid disease is a rare but serious occurrence. The positive diagnosis relies on multiple factors, including epidemiological, clinical, radiological and parasitological evidence. Both medical and surgical treatments play a crucial role in determining the patient’s prognosis. Long-term postoperative monitoring is essential to detect any recurrence and is primarily based on ultrasound, computed tomography and serology. The prevention is essentially based on interrupting the parasite’s life cycle, protecting livestock and controlling the dog population.

## Abbreviations

AE: alveolar echinococcosis
ALT: alanine transaminase
AST: aspartate transaminase
CE: cystic echinococcosis
CT: computed tomography
ELISA: enzyme-linked immunosorbent assay
IHA: indirect heamagglutination technique
US: ultrasound
WHO: World Health Organization
